# Patient-Reported Outcomes and Body Composition Changes in Patients With Colorectal Cancer During Chemotherapy: A Longitudinal Study

**DOI:** 10.1155/jonm/1268096

**Published:** 2025-08-24

**Authors:** Yuxuan Wu, Xinyuan Yu, Siting Huang, Xinchu Luo, Dandan Lv, Huimin Du, Jincheng Zhang, Xi Ke, Dun Liu

**Affiliations:** ^1^School of Nursing, Fujian Medical University, Fuzhou 350005, Fujian, China; ^2^Gastric Cancer Center, West China Hospital of Sichuan University, Chengdu 610095, Sichuan, China; ^3^Department of Gastroenterology, The Second Affiliated Hospital of Chongqing Medical University, Chongqing 400015, China; ^4^Cancer Bio-Immunotherapy Center, Fujian Provincial Cancer Hospital, Fuzhou 350005, Fujian, China

## Abstract

**Aims:** To investigate the changes in self-reported outcomes and body composition among colorectal cancer (CRC) patients over the course of chemotherapy and differences in body composition among different self-reported trajectory subgroups.

**Design:** Prospective longitudinal study.

**Methods:** This longitudinal study surveyed 201 CRC patients from a cancer hospital in Fuzhou, China. Collected via a patient-reported outcome questionnaire, a bioelectrical impedance analyzer, a grip strength meter, a 30-s standing and sitting test, body circumference measurements, and skinfold thickness measurements. We explored trends in the abovementioned variables in the early, middle, and late stages of chemotherapy in CRC patients and differences in body composition under different patient reporting trajectories. This study adhered to the relevant STROBE checklist-guided reporting.

**Results:** The overall severity of the chemotherapy results reported by the patients increased from T1 to T2 (overall health outcome: −7.51%; function: −2.55%; and symptoms: 0.67%), followed by a slow recovery (overall health outcome: 1.31%; function: 1.83%; and symptoms: −2.79%). Different trajectory subgroups were observed, indicating the complexity of the patients' experiences during chemotherapy. During chemotherapy, obesity-related indicators (weight, BMI, body fat rate, etc.) significantly increased (*p* < 0.05). Although skeletal muscle mass did not change significantly over time (*p* > 0.05), the overall outcome and symptom trajectory subgroups reported by the patients during chemotherapy (*p* < 0.05) were significantly different.

**Conclusions**: It is necessary for healthcare workers to conduct dynamic assessments of patients with moderate to severe self-reported results as early as possible, give attention to the deterioration of body composition during chemotherapy, and explore effective measures to improve patients' body compositions to further optimize the patient experience.

**Implications for Nursing Management:** Nursing managers should promptly monitor patients' body composition and subjective experiences changes during treatment. Customized supportive care based on body composition measurements to optimize health outcomes, enhancing the personalization and precision of medical services.

## 1. Introduction

Colorectal cancer (CRC) is a common malignant tumor of the gastrointestinal tract that ranks among the top three in terms of cancer-related morbidity and mortality worldwide. According to global cancer statistics [1], there were approximately 1.9 million new cases of CRC and 904,000 related deaths worldwide in 2022, accounting for nearly one-tenth of all cancer cases and cancer-related deaths. Chemotherapy is an effective method to improve the survival time of CRC patients, shrink tumor lesions, improve the success rate of surgery, eliminate micrometastases that may recur, improve the survival rate of patients with early- and middle-stage disease, alleviate symptoms [2], and enhance quality of life in patients with advanced CRC [3].

However, while chemotherapeutic drugs exert beneficial effects on CRC by killing tumor cells, they also cause varying degrees of symptom burden [4]. Some studies have shown that [5] approximately 60% of the patients with gastrointestinal tumors experience moderate-to-severe symptom burden during chemotherapy. Bonhof et al. [6] reported that fatigue, anxiety, depression, and sleep disturbances persist 1 year after the end of treatment in CRC patients undergoing chemotherapy. Chen and Xuan [7] reported that the symptom burden of CRC patients varies at different stages of chemotherapy. It is necessary to take effective measures to monitor changes in patient experience during chemotherapy. Patient-reported outcomes (PROs) can be used to evaluate physical function, mental and psychological health, social activities, and treatment from the patients' point of view, thereby better reflecting patients' experiences [8]. Lee et al. [9] reported that PROs are often more reliable and accurate than physician-reported outcomes and that they are more strongly correlated with clinical outcomes. The measurement of PROs is important for accurately assessing the physical and psychological experiences of cancer patients during treatment [8, 10]. At present, PROs have played a significant role in the evaluation of cancer treatment efficacy, adverse reaction monitoring, and disease outcome prediction [11]. However, few studies regarding the trajectory changes of patients with CRC during chemotherapy have been reported [12, 13], and most of these studies focus on a single PRO symptom or the overall difference in PROs over the course of chemotherapy without further capturing the heterogeneity of overall trajectory changes at different time sequences during chemotherapy. Therefore, it is necessary to conduct a dynamic analysis of the heterogeneity of PRO trajectories in patients with CRC during chemotherapy to further reveal the complex changes of PROs in the long-term treatment process.

In addition, chemotherapy may also lead to changes in the body composition of CRC patients. Dolly et al. [14] performed computed tomography (CT) and reported that CRC patients experienced loss of skeletal muscle mass, total adipose tissue mass, and bone mineral density after four cycles of chemotherapy. Yoon et al. [15] studied the use of bioelectrical impedance analyzers to measure PROs among gastrointestinal cancer patients and reported that chemotherapeutic agents accelerated the catabolism of proteins in skeletal muscle, leading to adverse outcomes for CRC patients. Lee et al. [16] reported that CRC patients receiving postoperative adjuvant chemotherapy were more likely to experience weight gain than weight loss during adjuvant chemotherapy. Thus, chemotherapy exacerbates the occurrence of adverse body composition changes in CRC patients. Studies have shown that [17, 18] the alterations in body composition caused by chemotherapy are related mainly to decreased food intake due to gastrointestinal adverse effects, decreased physical activity inspired by generalized fatigue, abnormal energy and fat metabolism, and the direct effects of chemotherapy on muscle.

Moreover, while chemotherapy exacerbates the loss of muscle mass in patients, the continued loss of muscle mass exacerbates chemotherapy-related toxicity because muscle is an important organ for drug distribution and metabolism, and patients with sarcopenia have higher concentrations of drugs in their bodies and are thus more prone to chemotherapy-related toxicity [18]. Baracos and Arribas [19] reported that the dual stress of loss of muscle mass and function with fat accumulation also leads to poor prognosis in cancer patients by exacerbating complications such as weakness, disability, metabolic disorders, and chemotherapy dose-limiting toxicity. Systemic inflammation, oxidative stress, and imbalances in the protein hydrolysis system are the primary mechanisms of skeletal muscle mass loss, leading to reduced force production, susceptibility to fatigue, and decreased exercise capacity, which can severely impact patients' quality of life [20, 21]. However, insufficient attention has been given to body composition changes during chemotherapy in CRC patients in the clinic, and body composition measurements are often expressed in terms of body weight and body mass index, which lack accuracy. Patients with CRC may have stable or increased body weight during chemotherapy due to an increase in the third interstitial fluid (e.g., ascites and lower limb edema), masking the deterioration of the patient's other body components [22].

To date, most studies regarding PROs and body composition have been conducted separately, with few studies focusing on the trajectory changes of both during the same round of chemotherapy. In addition, the overall body composition and changes in body composition in CRC patients during chemotherapy have not been monitored sufficiently in the clinic, and their impact on the patient experience is often not considered by clinicians. Therefore, the aims of this study are to (1) understand the longitudinal trajectory changes of PROs and body composition in patients with CRC at different stages of chemotherapy and (2) further explore whether there are differences in body composition among different trajectory subgroups of PROs in patients with CRC during chemotherapy.

## 2. Materials and Methods

### 2.1. Study Design and Setting

Patients with CRC who were seen and started chemotherapy at a tertiary class A cancer hospital in China from December 21, 2022, to December 31, 2023, were randomly selected for the study. In accordance with the Chinese Society of Clinical Oncology CRC Clinical Guidelines 2023 Edition [23], the current standard course of chemotherapy for treating CRC lasts for 36 months, with 8–12 cycles at intervals of 1-2 weeks. The data were collected at three time points: in the early stage of chemotherapy (within 1 month of chemotherapy and the 1st-2nd cycles of the chemotherapy cycle), in the middle stage of chemotherapy (1–3 months of chemotherapy and the 3rd‒6th cycles of chemotherapy), and in the late stage of chemotherapy (the 4th month of chemotherapy and above and the 7th‒12th cycles of chemotherapy).

1. Diagnostic criterion: CRC diagnosed by histopathology.
2. Inclusion and exclusion criteria

The inclusion criteria were as follows: ① patients indicated for chemotherapy; ② patients aged ≥ 18 years; ③ patients who were able to complete the required study questionnaire independently or with the help of the investigator; and ④ patients who were informed of their diagnosis and agreed to participate in the study.

The exclusion criteria were as follows: ① tumor recurrence or metastasis; ② cognitive dysfunction and inability to accurately express one's thoughts; ③ severe systemic infections or the combination of other serious physical illnesses such as limb dysfunction, ascites, anemia, infections, and cardiac disease; ④ history of other psychiatric disorders or central nervous system diseases; ⑤ receiving steroid therapy; ⑥ pregnancy; ⑦ receiving hospice care; and ⑧ peripheral edema, severe heart failure, abnormal liver and kidney function, severe hypoalbuminemia, metal implants, pacemakers, and any other diseases that affect the BIA measurement.

### 2.2. Ethics

All patients signed an informed consent form before participation. The study was approved by a medical research ethics review committee in Fujian Province (Approval no. 2021-98).

### 2.3. Research Tools

#### 2.3.1. Questionnaire on Demographic and Clinical Information

A homemade questionnaire was developed to assess demographic information, including sex, age, ethnicity, marital status, education level, occupation, employment status, economic level, payment method, and place of residence. In addition, the following basic clinical information was evaluated: disease diagnosis, pathological diagnosis, height, weight, body mass index, clinical stage, presence of comorbid chronic diseases, presence of other complications, adjuvant therapy, stoma type, site, chemotherapy regimen, cycle, pathological type, surgery status, presence of complications, red blood cell count, neutrophil ratio, lymphocyte ratio, hemoglobin, albumin, and potassium ion concentration.

#### 2.3.2. Core Quality-of-Life Questionnaire and CRC-Specific Quality-of-Life Scales (European Organization for Research and Treatment of Cancer (EORTC) QLQ-C30 and EORTC QLQ-CR29)

The QLQ-C30 is the core scale in the EORTC system of quality of life measurement scales for cancer patients [24] Chinese scholars Wan et al. [25] developed a Chinese version of the EORTC QLQ-C30 in 2005. The scale consists of 30 items across 15 dimensions, including 5 functional dimensions, 3 symptom dimensions, 6 specific items, and 1 overall health status dimension. In the QLQ-C30, the first 28 items are rated on a 4-point Likert scale ranging from 1 (“not at all”) to 4 (“very much”). The last two questions are rated on a 7-point scale ranging from 1 (“very poor”) to 7 (“very good”). The raw scores of the scales were linearly converted to 1–100 points via standardized calculation. Higher scores on the functional scales and overall health status represent better functional and overall health status whereas higher scores on the symptom scales and individual items represent more severe symptoms.

The EORTC QLQ-CR29 is a CRC-specific quality-of-life assessment scale that was revised by the EORTC in 2007 [26]. The self-reported QLQ-CR29 assesses 4 functional dimensions, including body intention, weight loss, body image, and sexual functioning problems, as well as 19 individual symptom entries. In 2017, Lin et al. [27] developed a Chinese version of the QLQ-CR-29, and its scoring standard is consistent with that of the QLQ-C30.

The combination of the EORTC QLQ-CR 29 and the EORTC QLQ-C30 has been widely used in clinical research and is suitable for the measurement of self-reported outcomes in patients with CRC [28]. Patients can typically complete the two scales within 15 min [29].^.^ The scoring methods are shown in Supporting Table 1.

#### 2.3.3. Body Composition Measurement

##### 2.3.3.1. Bioelectrical Impedance Body Composition Analysis

The assessment of body composition mainly includes direct assessment (in laboratory settings) and indirect assessment (in field settings). Common laboratory measurement methods include dual-energy X-ray absorptiometry (DXA), CT, and magnetic resonance imaging (MRI) [30]. CT is currently regarded as the gold standard for body composition measurement though it is not suitable for short-term follow-up and repeated measurements due to its time-consuming nature, radiation exposure, and cost issues [31]. DXA and MRI are also difficult to be widely used in clinical practice due to their high costs, long scanning and processing times, and the need for high-level technical training [31, 32]. Field measurement methods mainly include anthropometry (such as body mass index [BMI] and skinfold thickness), air displacement plethysmography, and bioelectrical impedance analysis (BIA). Among them, BIA is highly applicable in clinical practice due to its advantages of being rapid, portable, low-cost, highly repeatable, and noninvasive [32, 33]. BIA is a technique that estimates body composition by measuring the electrical resistance of the human body to weak currents. Currently, there are more than ten commonly used bioelectrical impedance analyzers, and there is no unified standard among patients with cancer [32]. Previous studies [34, 35] have reported that the accuracy of BIA measurement depends on the frequency technology of the instrument, population model, and standardized operation. Therefore, multiple frequencies are superior to a single frequency, appropriate critical values should be selected based on the population, and BIA measurement standardization should be followed to reduce measurement errors. Therefore, this study used a multifrequency bioelectrical impedance body composition analyzer (model: GS6.5C+), which adopts the original Chinese human body composition mathematical model and has been applicable for multiple sample tests in Chinese people since its development. This instrument has been used for research regarding sarcopenia and obesity in China [36, 37] and is effective, economical, and applicable. The GS6.5C+ measures the Appendicular Skeletal Muscle (ASM) of the limbs and defines the ratio of ASM to the square of height (m) as the Appendicular Skeletal Muscle mass Index (ASMI). ASM values < 7.0 kg/m^2^ in males and < 5.7 kg/m^2^ in females are defined as reduced skeletal muscle mass in the limbs, which is consistent with the Asia Working Group for Sarcopenia (AWGS) [38]. The BIA test process is simple, and the test time does not exceed 2 min. The measurements were conducted in accordance with the standardization of BIA measurement and at a constant temperature (18°C–22°C) at least 2 h before getting up or going to bed. The patients were instructed not to fast or receive intravenous infusion and to urinate and defecate prior to the measurement. The patients were also instructed to wear light clothing and remove metal accessories from the body. During measurements, the patients stood barefoot on the electrode, held the double-handle electrode rods with both hands with their arms appropriately extended at an angle of approximately 15° to the trunk, and stood still for the duration of the test.

##### 2.3.3.2. Skinfold Thickness

Measurement of cortical thickness using sebaceous forceps can reflect the body's subcutaneous fat and calculate the body fat percentage. The principle of calculating body fat percentage is based on determining the linear correlation between human body density and skinfold thickness, deducing the human body density and then substituting the human body density into the body fat percentage formula [39]. Researchers have used sebaceous pliers (type: LS0050) to measure the thickness of different parts of the skin folds to represent subcutaneous fat, which is accurate within 0.1 mm, and the average value of three measurements was used as the final measurement value.

##### 2.3.3.3. Body Circumference Measurement

Body circumference reflects the circumference of a cross-section of the body. The changes in skin, fat, and muscle in the cross section directly affect the size of the body circumference value and then affect the changes in the body shape. In this study, waist circumference, hip circumference, thigh circumference, upper arm circumference, and the waist‒hip ratio were selected as indicators of the body shape.

#### 2.3.4. Muscular Strength Measurements

##### 2.3.4.1. Upper Limb Muscle Strength

A grip strength meter (type: EH101) was used to measure upper limb muscle strength. The measurement procedure was as follows: the subject was standing with feet and shoulders level, his or her hands were naturally hanging down, and the hands were not close to the body. The patient squeezed the force gauge of the hand-held dynamometer with his or her maximum strength, for at least 5 s each time using his or her dominant hand. The grip strength measurements were repeated twice, and the maximum value was recorded.

##### 2.3.4.2. Lower Extremity Muscle Strength

The 30-s chair–stand test (30-s CST) was used as a reliable and valid indicator of lower extremity strength [40]. The test procedure was as follows: a chair (approximately 43 cm in height) was prepared and placed against a wall, and the subject sat in the middle of the chair with his or her arms crossed in front of his or her chest. Their backs were straight, and their feet were approximately shoulder width apart. When instructed to “start,” the subject repeated the sit‒stand cycle. The number of times the participant could complete the sit‒stand cycles within 30 s was recorded, and more repetitions indicated better lower extremity muscle strength.

### 2.4. Data Collection

Prior to data collection, the participants were informed of their right to decline participation in the study, the confidential nature of their participation, as well as their right to withdraw at any time. All participants provided written informed consent. The participants then completed demographic and disease characteristic questionnaires at the baseline, followed by three follow-up assessments of self-reported outcomes, body composition measurements, and muscle strength measurements in the early (< 1 month), middle (1–3 months), and late (≥ 4 months) stages of chemotherapy. Each data collection was conducted face-to-face by the researchers with the participants.

### 2.5. Statistical Methods

Data analysis was performed via SPSS 27.0, Origin 2021, and Mplus 7.0. Continuous variables that were normally distributed are expressed as the mean ± standard deviation; continuous variables that were not normally distributed are expressed as the median (interquartile spacing); and categorical variables are expressed as frequencies (percentages). Repeated-measures ANOVA was used for the data conforming to a normal distribution and homogeneity of variance. The generalized estimation equation and the Kruskal‒Wallis nonparametric test are used for the data that do not satisfy the normal distribution or the homogeneity test of variance. In addition, principal component analysis was used to downsize the high-dimensional data of the body components via Origin 2021 software. Mplus 7.0 software was used to perform trajectory analysis by calculating the Akaike information criterion (AIC), Bayesian information criterion (BIC), sample size-adjusted Bayesian information criterion (aBIC), entropy value, bootstrap likelihood ratio test (BLRT) value, and Lo‒Mendell‒Rubin test (LMR) value. Lower AIC, BIC, and aBIC values indicate better model fit. The entropy value ranges from 0 to 1, and a value higher than 0.8 is preferred. When the *p* values of the LMR and BLRT are significant (*p* < 0.05), the model with *k* categories is considered better than the model with *k* − 1 categories [41]. Differences were considered statistically significant at *p* < 0.05.

## 3. Results

### 3.1. Follow-Up

Data were collected from December 2022 to September 2023, and follow-up was carried out until December 2023. CRC patients who were within 1 month of starting chemotherapy (T1) and had received only 1-2 cycles of chemotherapy were initially enrolled, accounting for 241 patients. A second follow-up was conducted within 1–3 months of the patient's chemotherapy (T2), accounting for 218 patients. A third follow-up was conducted after the patient's chemotherapy had been administered for 4 months (T3), accounting for a total of 201 patients. The overall loss to follow-up rate in this study was 16.5%. The reasons for loss to follow-up are shown in Supporting Figure 1.

### 3.2. Baseline Demographic and Clinical Characteristics

The demographic and disease-related data of all 201 patients who completed the follow-up were analyzed. There were 115 (57.2%) men. The mean age was 56.85 ± 10.58 years, and the ages ranged from 24 to 80 years. There were no significant differences in the basic characteristics of the patients who were lost to follow-up and those who were not lost to follow-up (all *p* > 0.05) (shown in Table 1).

### 3.3. Trends in PROs

#### 3.3.1. Variability Analysis of PROs Over Time

Over the course of chemotherapy, there was a statistically significant difference in the overall change in self-reported emotional functioning, cognitive functioning, body image, quality of life, fatigue, constipation, blood and mucus in stools, abdominal pain, dry mouth, and hair loss between the stages of chemotherapy (all *p* < 0.05), as detailed in Table 2.

#### 3.3.2. Analysis of Overall Trends in PROs

**Figure 1:**
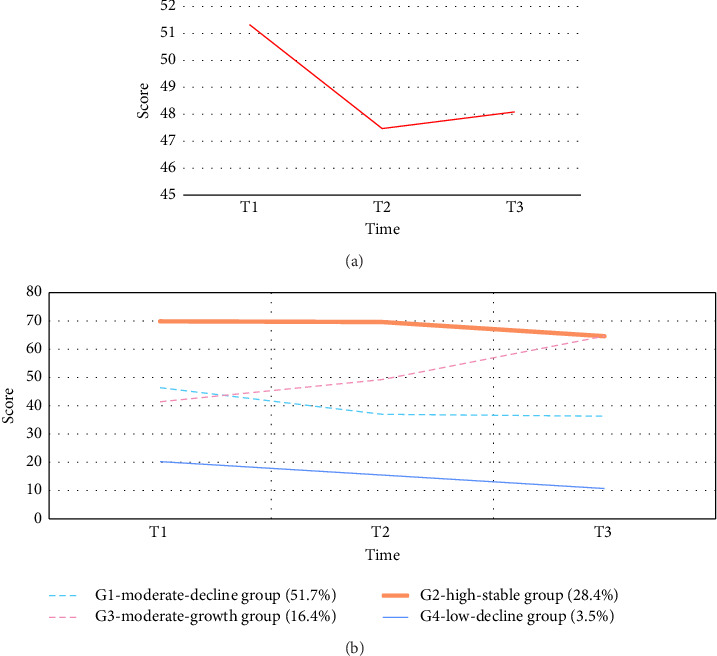
Trends and trajectories of changes in self-reported overall outcomes during chemotherapy for patients with CRC (a) trends in self-reported overall outcomes are shown. (b) Trajectories of change in potential categories of self-reported overall outcomes are shown.

An analysis of the overall trend of self-reported outcomes for CRC patients revealed that patients' self-reported quality-of-life scores first decreased from T1 to T2 (−7.51%) and then slowly recovered from T2 to T3 (1.31%), resulting in a downward trend from T1 to T3 (−6.29%) (Figure 1(a)).

Trajectory analysis was performed to examine the self-reported quality-of-life scores during chemotherapy on the basis of the fit indices. When the free estimation of the four-category model was chosen, the entropy value was greater than 0.7 (0.77), the LMR and BLRT values were significant (*p* < 0.05), the AIC (5102.35) and BIC (5148.60) were low, and the classification of the lowest probability of the category met the minimum requirements. The detailed results of the model parameters are shown in Supporting Table 2. According to the intercept and slope, the first category was the “moderate-decline group (G1),” which had the largest number of patients, with 104 patients (51.7%); the second category was the “high-stable group (G2),” with 57 patients (28.4%); the third category was the “moderate-growth group (G3),” with 33 patients (16.4%); and the fourth category was the “moderate-growth group (G4),” with only 7 patients (3.5%) (see Figure 1(b) for details).

#### 3.3.3. Trends and Trajectory Analysis of Self-Reported Functioning Outcomes

**Figure 2:**
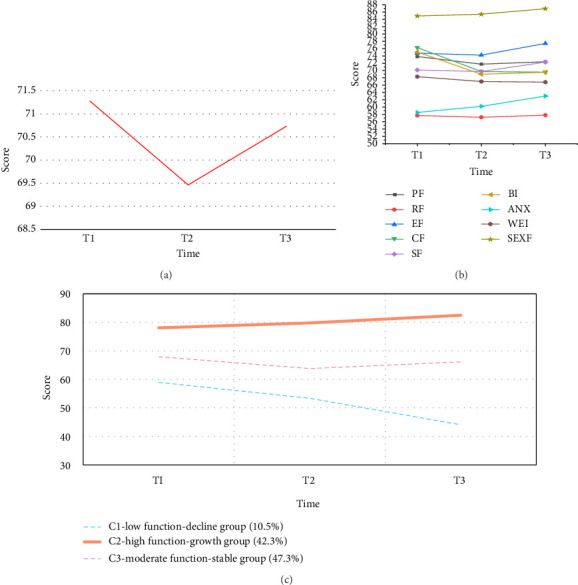
Trends and trajectories of changes in self-reported functional outcomes in patients with CRC during chemotherapy (a) overall trends in self-reported functioning outcomes are shown. (b) Trends in self-reported functioning outcomes across domains are shown. (c) Trajectories of changes in potential categories of self-reported functioning outcomes are shown. Note: T1 is the early stage of chemotherapy; T2 is the middle stage of chemotherapy; T3 is the late stage of chemotherapy; PF: physical functioning; RF: role functioning; EF: emotional functioning; CF: cognitive functioning; SF: social functioning; BI: body image; ANX: anxiety; WEI: weight; SEXF: sexual interest.

The mean self-reported functioning scores showed an overall decreasing trend from T1 to T2 (−2.55%) and a slow recovery from T2 to T3 (1.83%), resulting in a downward trend from T1 to T3 (−6.29%). The trajectory of change in each functional dimension revealed the lowest patient-reported role functioning scores, the highest reported sexual functioning scores, and the greatest change in body intention functioning (see Figures 2(a) and 2(b) for an illustration).

According to the fit indices, when the three categories of free estimation were selected, the entropy value was greater than 0.7 (0.83), the LMR and BLRT values were significant (*p* < 0.05), and the AIC (4672.83) and BIC (4709.17) were lower. In addition, the classification of the least-category probability met the minimum requirements. The detailed results of the model parameters are shown in Supporting Table 3. On the basis of the intercept and slope, the first category was named the “low function-decline group (C1)” and had the smallest number of patients (21 patients, accounting for 10.5% of the sample); the second category was named the “high function-growth group (C2),” with 85 patients (42.3%); and the third category was named the “moderate function-stable group (C3),” with 95 patients (47.5%) (Figure 2(c)).

#### 3.3.4. Analysis of Trends and Trajectories in Self-Reported Symptoms

**Figure 3:**
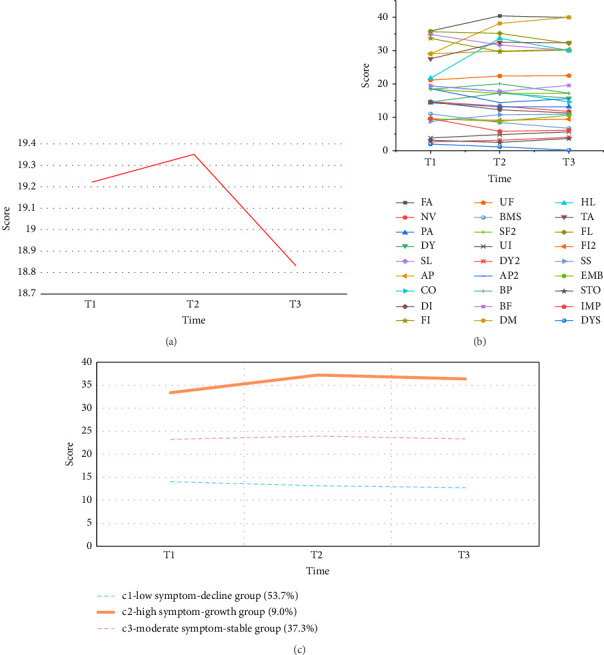
Trends and trajectories of changes in self-reported symptoms during chemotherapy for patients with CRC (a) overall trends in self-reported symptoms are shown. (b) Trends in self-reported symptoms across domains are shown. (c) Trajectories of change in potential categories of self-reported symptoms are shown. Note: T1 is early chemotherapy; T2 is middle-stage chemotherapy; T3 is late chemotherapy; FA: fatigue; NV: nausea/vomiting; PA: pain; DY: dyspnea; SL: sleep disturbance; AP: appetite loss; CO: constipation; DI: diarrhea; FI: financial problems; UF: urinary frequency; BMS: blood and mucus in the stool; SF2: stool frequency; UI: urinary incontinence; DY2: dysuria; AP2: abdominal pain; BP: buttock pain; BF: bloating; DM: dry mouth; HL: hair loss; TA: taste; FL: flatulence; FI2: fecal incontinence; SS: sore skin; EMB: embarrassment; STO: stoma care problems; IMP; impotence (male); DYS: dyspareunia (women).

Patient self-reported symptoms showed an overall general trend of change, with an increasing trend from T1 to T2 (0.67%), and then a gradual decrease from T2 to T3 (−2.79%), resulting in a downward trend from T1 to T3 (−2.03%). The trajectory of change in each symptom dimension yielded the highest patient-reported fatigue score, the lowest reported sexual pain score, and the greatest change in patient-reported dry mouth. The details are shown in Figures 3(a) and 3(b).

According to the fit indices, when the free estimation of the three-category model was chosen, the entropy value (0.81) was greater than 0.7, the LMR and BLRT values were significant (*p* < 0.05), the AIC (4157.95) and BIC (4194.29) were low, and the least-category probability classification met the minimum requirements. The detailed results of the model parameters are shown in Supporting Table 4. On the basis of the intercept and slope, the first category was the “low symptom-decline group (c1),” which had the largest number of patients (108 patients, accounting for 53.7% of the sample); the second category was the “high symptom -growth group (c2),” which had the lowest number of patients (18 patients, 9.0%); and the third category was the “moderate symptom-stable group (c3),” with 75 patients (37.3%) (Figure 3(c)).

### 3.4. Analysis of Trends and Compositional Categories of Body Composition Changes During Patient Chemotherapy

#### 3.4.1. Overall Trends in the Values of Each Indicator of Patient Body Composition

The results revealed that body weight; body mass index; body fat content; waist-to-hip fat ratio; visceral fat content; chest circumference; waist circumference; hip circumference; upper arm circumference; thigh circumference; and the skin fold thickness of the triceps, scapular, abdominal, and anterior iliac spaces significantly increased over the course of chemotherapy, and the difference between timepoints was statistically significant (*p* < 0.05), as detailed in Table 3.

**Figure 4:**
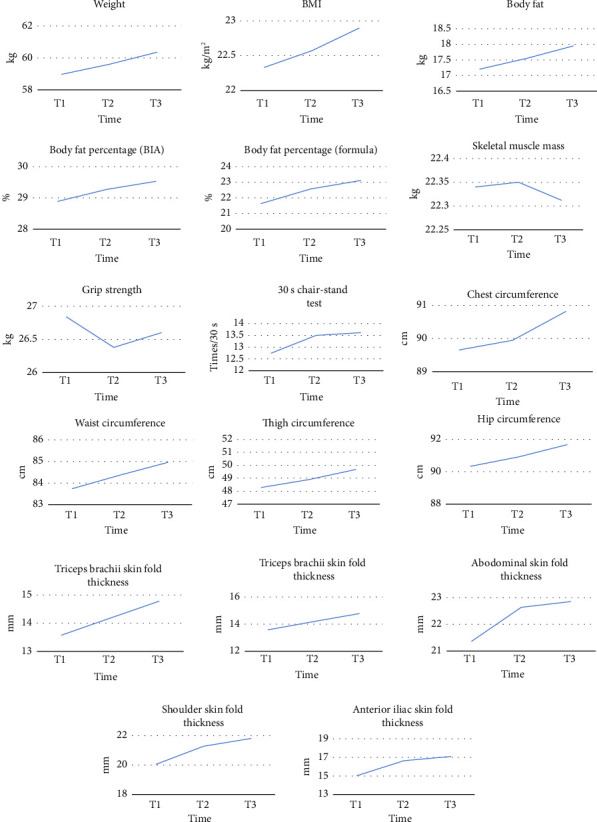
Changes in body composition indicators at three time points during chemotherapy for colorectal cancer.

An analysis of the trends of each body composition variable revealed that all body composition indicators had an increasing trend, except for the patients' skeletal muscle mass and upper limb grip strength, which showed a decreasing trend (Figure 4).

#### 3.4.2. Principal Component Dimensionality Reduction Analysis of Patient Body Composition

**Figure 5:**
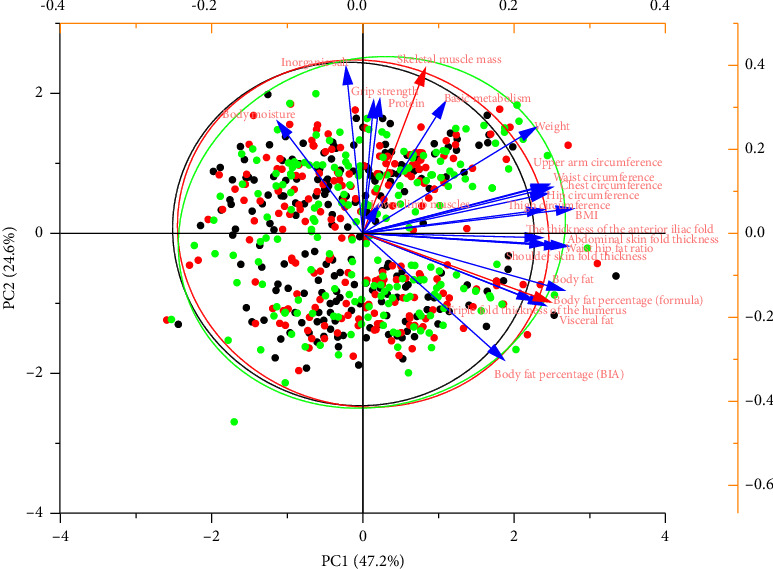
Principal component analysis results of various body composition indicators.

Principal component analysis downscaling of the body composition indicators revealed that the cumulative proportion of variance explained by the first two principal components was 71.8%. Furthermore, the body fat percentage (PC1 = 0.248), which represents the fat content, and the skeletal muscle mass (PC2 = 0.392), which represents the fat-free content, were mutually independent and the most representative measures of body composition, as detailed in Figure 5.

### 3.5. Analysis of the Variability of Study Participants' Body Composition Across Trajectories of Self-Reported Quality of Life, Functioning Outcomes, and Symptom Development

Table 4 shows the analysis of the variability of skeletal muscle mass scores across the developmental trajectory subgroups of self-reported outcomes for CRC chemotherapy patients at different stages of chemotherapy. The results of the Kruskal‒Wallis H test revealed that there was a statistically significant difference in skeletal muscle mass among the three periods, T1, T2, and T3 (all *p* < 0.05). Furthermore, in the between-group comparison, skeletal muscle mass was significantly greater in the high-stable group (G2) than that in the moderate-decline group (G1) and significantly greater in the moderate-growth group (G3) than that in the moderate-decline group (G1) (*p* < 0.05). Finally, there was a significant difference in skeletal muscle mass at T2 and T3 across symptomatic trajectory subgroups (both *p* < 0.05), with the low symptom-decline group (c1) having a greater skeletal muscle mass than the high symptom-growth group (c2) and the moderate symptom-stable group (c3).

Table 5 shows the analysis of the variability of body fat percentage in CRC chemotherapy patients' scores across developmental trajectory subgroups of self-reported outcomes for different chemotherapy durations, and the results of the Kruskal‒Wallis H test revealed that none of the differences in body fat percentage across chemotherapy durations were statistically significant across the different subgroups of development of PROs (all *p* > 0.05).

## 4. Discussion

In this study, we found that PROs among CRC patients tended to exacerbate, followed by remission during chemotherapy, but there were subgroups with different developmental trajectories. Body composition indices, especially adiposity indices, increased significantly over time during chemotherapy. The skeletal muscle masses of different PRO development trajectory groups differ. These findings are important because they provide a new perspective for improving the outcomes of patients undergoing chemotherapy for CRC.

First, during chemotherapy, the self-reported overall outcomes (−7.5%), functions (−2.55%), and symptoms (0.67%) increased and then recovered slowly (overall outcomes: 1.31%; functions: 1.83%; and symptoms: −2.79%). Such an overall trend reflects the average change in the subjective experience of chemotherapy among the study population; the adverse reactions caused by chemotherapy gradually worsened from the early stage (within 1 month) to the middle stage (1–3 months) of chemotherapy. It may be necessary to take preventive measures in advance, such as symptomatic treatment and patient education. By the late stage of chemotherapy (after 4 months), the adverse reactions are resolved, and the subsequent treatment plan and nursing plan can be adjusted accordingly. Understanding the overall trend change can provide a reference for the formulation of overall treatment and nursing strategies for patients with CRC who are undergoing chemotherapy. Scheepers et al. [42] reported that the quality of life in CRC patients who received adjuvant chemotherapy after surgery recovered more slowly during postoperative chemotherapy, and there was a significant decline in quality of life from the baseline to 3 months, with a gradual recovery after 3 months and a return to baseline levels within 12 months. This trend may be related to early organismal chemotherapy intolerance and increased tension, anxiety, and psychological stress in the early stage of chemotherapy. Second, based on the results of the potential class growth model analysis, the overall condition, functional status, and symptoms reported by patients with during chemotherapy showed different heterogeneous change trajectories within the patient group, reflecting the heterogeneity within the patient population. Although the change trajectories of G1 (accounting for 51.7%), C3 (accounting for 47.3%), and c3 (accounting for 37.3%) in the trajectory grouping were similar to their corresponding overall trends, this group accounted for a large proportion of the total population. This large overall trend made it easier for medical staff to overlook G4 (accounting for 3.5%), C1 (accounting for 10.5%), and c2 (accounting for 9.0%) who reported severe outcomes and deteriorated during chemotherapy. Therefore, while understanding the changes in the overall trends, medical staff should also strive to identify minority patients who have a high risk of having severe adverse reactions as early as possible so that the treatment plan can be personalized. In addition, the mechanisms and influencing factors of these results must be investigated to provide more targeted intervention measures to achieve more precise and effective personalized medical care.

In terms of body composition, in this study, weight, BMI, body fat content, body fat percentage, body circumference, and skinfold thickness, which represent the fat content, significantly increased over the course of chemotherapy (*p* < 0.05). Kenkhuis et al. [43] reported that approximately 44% of the CRC patients were overweight and 31% were obese during chemotherapy, and these values significantly increased within 24 months of treatment. Although this study did not measure the dietary intake and physical activity of patients during chemotherapy, previous studies have reported the significance of exercise, dietary adjustments, and nutritional therapy for weight control and obesity during treatment, which may in turn affect the development and treatment outcomes of CRC [44, 45]. A previous study [46] reported that the correlation curve between body weight and cancer outcomes is U shaped, with both underweight and overweight patients having poorer outcomes. Lee et al. [47] reported that although weight gain (≥ 5 kg) during chemotherapy for CRC was not associated with disease-free survival during treatment, weight gain (≥ 5 kg) was significantly associated with poorer prognosis in overweight and obese patients. This is related to the fact that obesity exacerbates the inflammatory response of the body, alters hormone levels, and leads to dysbiosis of the intestinal flora, among other mechanisms, thereby aggravating the development of CRC [48]. Therefore, measurements of body composition during chemotherapy are necessary for patients with CRC, and the impact of obesity on patients undergoing chemotherapy for CRC still needs to be further investigated. We further propose that subsequent studies should focus on dietary modifications tailored to individual body composition profiles (e.g., high-calorie and high-protein diets) and consider patients' exercise preferences to recommend specific exercise regimens (e.g., aerobic or resistance training), thereby optimizing body composition and mitigating chemotherapy-related adverse effects. Such investigations would enable the formulation of early, personalized interventions customized to patients with diverse body compositions.

Moreover, in the present study, water, protein, inorganic salt, and skeletal muscle contents, which represent fat-free contents, did not change significantly during chemotherapy (*p* > 0.05), which is inconsistent with the results of other studies. Pedrosa et al. [49] reported that chemotherapy exacerbates skeletal muscle atrophy in cancer patients and that the loss of skeletal muscle may further exacerbate cancer-associated cachexia with systemic multifactorial syndromes of systemic inflammation, metabolic disorders, anorexia, and insulin resistance, exacerbating patient fatigue and decreasing quality of life. At present, significant changes in fat-free mass have not been observed during chemotherapy. This may be due to the fact that the skeletal muscle mass of the patients in this study was relatively low before chemotherapy, the follow-up period was short, or the accuracy and precision of BIA in body composition measurement were not satisfactory. As an economical, rapid, easy-to-use, and radiation-free indirect method for measuring human body composition, BIA has been accepted as one of the reliable methods for assessing sarcopenia by European [50] and Asian sarcopenia guidelines [38] as well as the international consensus [51] on cachexia. However, due to its reliance on several physical assumptions in measurement and its potential to be affected by parameters such as hydration, skin temperature, sweat, and bladder fullness, BIA may overestimate fat-free mass compared with more complex methods such as DXA, CT, or MRI [52]. Therefore, BIA measurement results should be interpreted with caution. Caan et al. [53] reported that patients with sarcopenia had a 27% greater overall risk of death than nonsarcopenic patients did over a mean follow-up period of 5.8 years and that female patients with low muscle and high levels of obesity had a 64% greater overall risk of death than obese women with adequate musculature. It has also been shown [54] that sarcopenia is associated with a significantly worse overall survival prognosis and dose-limiting toxicity in patients with metastatic colon cancer receiving chemotherapy. Therefore, more attention should be given to CRC patients with abnormal skeletal muscle mass during chemotherapy to provide targeted interventions for patients with sarcopenia.

Finally, in this study, there were significant intergroup differences in skeletal muscle mass among the subgroups of patient-reported trajectories of overall health outcomes (all *p* values < 0.05). Patients in group G2, who had higher and more stable overall health outcome scores from T1 to T3, had better skeletal muscle mass compared with patients in groups G1 and G3 with moderate scores. Moreover, in the symptom trajectory grouping, patients with lower symptom scores at T2 and T3 (c1) had higher skeletal muscle mass than those with moderate or high symptoms (*p* < 0.05). These results indicate that the patient-reported symptoms, overall health status, and skeletal muscle mass are significantly correlated during chemotherapy. The related mechanisms also provide an indirect explanation for the possible synergistic effect between the adverse experiences caused by chemotherapy drugs and the reduction in skeletal muscle mass. Previous studies [55, 56] have reported that the reduction in skeletal muscle content can alter the distribution, metabolism, and clearance rate of chemotherapy drugs, leading to an increase in toxic adverse effects of chemotherapy. Common chemotherapy drugs are used to inhibit muscle protein synthesis, reduce myogenic differentiation, activate muscle protein degradation, increase oxidative stress, and reduce mitochondrial biogenesis, ultimately causing skeletal muscle atrophy or emaciation [57]. Therefore, chemotherapy may exacerbate muscle atrophy. The higher the dose of chemotherapy distributed in the skeletal muscles, the more severe the toxic side effects will be, and the more severe the adverse experiences of the patients will be. Another study [58] reported a lower muscle volume in patients with breast cancer resulting in a smaller tissue volume for chemotherapeutic drug distribution, with potentially reduced metabolism and drug clearance, leading to greater toxicity. Sousa et al. [59] reported that alterations in body composition during the treatment of gastrointestinal cancer patients and patient-reported weakness and incapacity may have similar mechanisms of occurrence, such as loss of body muscle tissue, decreased strength and physical function, low food intake, and organismal inflammation. This finding also suggests that there may be a potential synergistic effect between PROs and body composition. Therefore, future studies should focus on the interaction and synergy mechanisms between skeletal muscles and PROs and continuously assess and measure the trends and correlations between PROs and skeletal muscles in patients with CRC during chemotherapy. The optimal timing and treatment strategies for preventing or delaying the abnormal development of muscles should also be explored to improve the chemotherapy experience of patients with CRC.

### 4.1. Limitations

There are several limitations in this study. First, considering the clinical reality and difficulty of survey implementation, data related to PROs and body composition before chemotherapy were not collected in the longitudinal follow-up of this study. Second, considering the feasibility of survey implementation, this study was conducted only in one hospital and did not include patients from hospitals in other regions and grades, which may decrease the representativeness of the results. Third, this study used the BIA indirect measurement method to collect body composition, which may underestimate body fat and overestimate skeletal muscle mass. Finally, our assessment of PROs was in the form of self-report questionnaires, which could overestimate or underestimate the real results. In future research, a combination of subjective patient reports and objective indicators of tumor outcomes can be used to estimate chemotherapy efficacy, more validation tools can be added to the body composition measurements of patients with CRC, multicenter surveys with larger sample sizes can be conducted, and the follow-up period can be increased to validate the results of this study.

## 5. Conclusions

During chemotherapy, the PROs of patients with CRC were most severe in the early to middle stages. However, the trajectories for PROs differed between patient groups, and it is necessary to continue analyzing the characteristics of the changes in trajectories of different PROs heterogeneity groups, identify patients with severe high-risk abnormalities in PROs during chemotherapy, and conduct dynamic assessments as early as possible. During chemotherapy, the patient weight increased (2.36%), and the increase in body fat (4.36%) and BMI (2.55%) must be controlled. Finally, patients with low skeletal muscle mass were more likely to appear in the poorer patient-reported overall outcomes and high symptom groups. These changes suggest that providers must pay attention to the internal heterogeneity within the group (early identification of high-risk groups and personalized intervention) and the impact of body composition on tumor outcomes. It is recommended that a healthy body composition be maintained through nutritional intervention (such as increasing protein ratio and limiting calories) and exercise (aerobic and resistance exercises), optimizing the cancer treatment and rehabilitation experience of patients with CRC.

## 6. Implications for Nursing Management

Our findings indicate that patients with CRC may experience corresponding changes in self-reported symptoms, function, and quality-of-life outcomes and body composition during chemotherapy. Patients who report more severe symptoms and quality-of-life burdens may also have poor body composition (such as low skeletal muscle mass), which suggests a potential synergistic effect between PROs and body composition analysis. First, nursing managers should give attention to patients' subjective feelings in the clinical process rather than relying solely on objective indicators. PROs should be incorporated into routine clinical practice as soon as possible, further strengthening the concept of the “patient-centered, disease chain”. Second, tumors are chronic diseases, and individuals with CRC require long-term lifestyle interventions and medication treatment to delay the progression of the disease or even reverse its outcome. Studies have shown [60] that implementing exercise and nutritional interventions can prevent and reverse patients' body composition, which may improve their overall health status and survival rate. Changes in body composition, such as skeletal muscle and body fat, are related to the prognosis of cancer patients [61], and interventions such as exercise and nutrition may improve patients' body composition. BIA has been widely used as the most convenient screening method for measuring body composition [62]. Nursing managers should give attention to the measurement of body composition upon admission. It is necessary to measure the body composition of patients upon admission and develop and provide customized supportive care on the basis of the personalized information provided by the measurement results. While monitoring relevant objective physiological indicators, dynamic monitoring of PROs should be carried out to ensure the safety and effectiveness of the entire treatment and rehabilitation process, ultimately achieving the goal of optimizing patient health outcomes, making medical services more personalized and accurate, and gradually improving the quality of nursing services.

## Figures and Tables

**Table 1:** Comparison of the demographic and disease profile characteristics of patients who completed follow-up and those who were lost to follow-up (patients (percentages, %)).

Sports event		Completed follow-up (*n* = 201 cases)	Lost to follow-up (*n* = 40 cases)	*χ* ^2^	*p*
Sex	Male	115 (57.2)	26 (65.0)	0.83	0.39
	Female	86 (42.8)	14 (35.0)		

Age (years)	1 < 30	3 (1.5)	0 (0.0)	5.51	0.32
	30–40	8 (4.0)	3 (7.5)		
	41–50	38 (18.9)	4 (10.0)		
	51–60	78 (38.8)	14 (35.0)		
	61–70	60 (29.9)	13 (32.5)		
	71–80	14 (7.0)	6 (15.0)		

Marital status	Married	194 (96.5)	40 (100.0)	1.44	0.66
	Unmarried	3 (1.5)	0 (0.0)		
	Divorcee	3 (1.5)	0 (0.0)		
	Bereft of one's spouse	1 (0.5)	0 (0.0)		

Education	Junior high school and below	137 (68.2)	32 (80.)	2.80	0.72
	Vocational secondary school	22 (10.9)	3 (7.5)		
	Senior high school	22 (10.9)	3 (7.5)		
	College	8 (4.0)	2 (5.0)		
	Undergraduate	9 (4.8)	0 (0.0)		
	Bachelor's degree	3 (1.5)	0 (0.0)		

Employment	Employed	47 (23.4)	6 (15.0)	1.45	0.48
	Laid off/unemployed	119 (59.2)	27 (67.5)		
	Retired	35 (17.4)	7 (17.5)		

Area of residence	Urban	47 (23.4)	13 (32.5)	1.98	0.52
	Rural	37 (18.4)	8 (20.0)		
	Other areas of the province	116 (57.7)	19 (47.5)		
	Out of province	1 (0.5)	0 (0.0)		

Monthly living expenses (yuan)	< 1000	4 (2.0)	4 (10.0)	5.97	0.15
	1000–3000	91 (45.3)	17 (42.5)		
	3000–8000	92 (45.8)	18 (45.0)		
	8000–15,000	12 (6.0)	1 (2.5)		
	> 15,000	2 (1.0)	0 (0.0)		

Smoking status	Smoking	32 (15.9)	9 (22.5)	1.02	0.36
	None/quit smoking	169 (84.1)	31 (77.5)		

Tumor site	Rectum	73 (36.3)	21 (52.5)	4.61	0.09
	Colon	117 (58.2)	19 (47.5)		
	Rectocolonic junction cancer	11 (5.5)	0 (0.0)		

Degree of differentiation	Low polarization	18 (9.0)	9 (22.5)	5.20	0.07
	Middle ground	177 (88.1)	30 (75.0)		
	High differentiation	6 (3.0)	1 (2.5)		

Clinical staging	Stage I	2 (1.0)	0 (0.0)	1.93	0.60
	Stage II	14 (7.0)	3 (7.5)		
	Stage III	60 (29.9)	16 (40.0)		
	Stage IV	125 (62.2)	21 (52.5)		

Number of chronic diseases combined	0	144 (71.6)	24 (60.0)	4.37	0.11
	1	42 (20.9)	9 (22.5)		
	≥ 2	15 (7.5)	7 (17.5)		

Other complementary therapies	Chinese medicine	10 (5.0)	2 (5.0)	6.64	0.13
	Radiotherapy	62 (30.8)	5 (12.5)		
	Target	12 (6.0)	3 (7.5)		
	Immunity (disease)	2 (1.0)	0 (0.0)		
	None	115 (57.2)	30 (75)		

Surgeries	No operation	77 (38.3)	16 (40.0)	0.96	0.63
	Postradical surgery	112 (55.7)	20 (50.0)		
	Postpalliative	12 (6.0)	4 (10.0)		

Radiotherapy	FOLFIRI	26 (13.0)	1 (2.5)	9.68	0.07
	FOLFOX	61 (30.3)	13 (32.5)		
	FOLFOXIRI	38 (18.9)	3 (7.5)		
	XELOX	60 (29.9)	18 (45.0)		
	RALOX	6 (3.0)	2 (5.0)		
	Other programs	10 (5.0)	3 (7.5)		

Stoma	None	183 (91.0)	37 (92.5)	1.10	0.76
	Colostomy	15 (7.5)	3 (7.5)		
	Rectal stoma	3 (0.15)	0 (0.0)		

*Note:* FOLFIRI regimen: irinotecan + 5⁃FU + aldesmethylfolate; FOLFOX regimen: oxaliplatin + 5⁃FU + aldesmethylfolate; FOLFOXIRI regimen: oxaliplatin + irinotecan + 5⁃FU + aldesmethylfolate; XELOX regimen: capecitabine + oxaliplatin; RALOX regimen: oxaliplatin + raltitrexed.

**Table 2:** Comparison of self-reported quality of life scores among patients with colorectal cancer at different timepoints (score, M (P25, P75)).

Dimension (math.)	T1 (*n* = 201)	T2 (*n* = 201)	T3 (*n* = 201)	*F*/*χ*^2^	*p*
*Global Health Status*					
Quality of life	50.00 (33.33, 66.67)^∗^	41.67 (33.33, 58.33)	50.00 (33.33, 66.67)	8.27	0.02

*Function Score*					
Physical functioning	80.00 (60.00, 93.33)	73.33 (60.00, 86.67)	73.33 (60.00, 86.67)	2.72	0.26
Role functioning	66.67 (33.33, 66.67)	50.00 (41.67, 66.67)	66.67 (33.33, 66.67)	0.12	0.94
Emotional functioning	83.33 (58.33, 91.67)	83.33 (58.33, 91.67)^∗^	83.33 (58.33, 95.83)	6.90	0.03
Cognitive functioning	83.33 (66.67, 100.00)^∗^	66.67 (50.00, 83.33)	66.67 (50.00, 83.33)	25.23	< 0.01
Social functioning	66.67 (50.00, 83.33)	66.67 (50.00, 83.33)	66.67 (50.00, 83.33)	3.52	0.17
Body image	77.78 (66.67, 100.00)^∗^	77.78 (55.56, 88.89)	66.67 (55.56, 88.89)	9.43	0.01
Anxiety	66.67 (33.33, 100.00)	66.67 (33.33, 66.67)	66.67 (33.33, 66.67)	3.94	0.14
Weight	66.67 (33.33, 100.00)	66.67 (33.33, 100.00)	66.67 (33.33, 100.00)	0.43	0.81
Sexual interest	100.00 (66.67, 100)	100.00 (66.67, 100.00)	100.00 (66.67, 100.00)	1.95	0.40
Composite function score	71.94 (62.31, 81.64)	69.06 (60.14, 79.39)	72.39 (63.86, 81.39)	4.84	0.09

*Symptom Score*					
Fatigue	33.33 (22.22, 55.56)^∗^	33.33 (22.22, 55.56)	33.33 (22.22, 55.56)	7.25	0.03
Nausea/vomiting	0.00 (0.00, 33.33)	0.00 (0.00, 16.67)	0.00 (0.00, 16.67)	5.06	0.08
Pain	0.00 (0.00, 16.67)	0.00 (0.00, 16.67)	0.00 (0.00, 16.67)	0.91	0.63
Dyspnea	0.00 (0.00, 33.33)	0.00 (0.00, 33.33)	0.00 (0.00, 33.33)	3.31	0.19
Sleep disturbance	33.33 (0.00, 66.67)	33.33 (0.00, 66.67)	33.33 (0.00, 66.67)	1.64	0.20
Appetite loss	33.33 (0.00, 66.67)	33.33 (0.00, 66.67)	33.33 (0.00, 66.67)	0.32	0.85
Constipation	0.00 (0.00, 33.33)^∗^	0.00 (0.00, 33.33)	0.00 (0.00, 33.33)	6.65	0.04
Diarrhea	0.00 (0.00, 33.33)	0.00 (0.00, 33.33)	0.00 (0.00, 33.33)	3.08	0.22
Financial problems	33.33 (0.00, 66.67)	0.00 (0.00, 66.67)	33.33 (0.00, 66.67)	2.84	0.24
Urinary frequency	16.67 (0.00, 33.33)	16.67 (0.00, 33.33)	16.67 (0.00, 33.33)	0.83	0.66
Blood and mucus in stools	0.00 (0.00, 16.67)^∗^	0.00 (0.00, 16.67)	0.00 (0.00, 16.67)	19.93	< 0.01
Stool frequency	16.67 (0.00, 33.33)	16.67 (0.00, 25.00)	0.00 (0.00, 33.33)	0.74	0.69
Urinary incontinence	0.00 (0.00, 0.00)	0.00 (0.00, 0.00)	0.00 (0.00, 0.00)	2.65	0.27
Diarrhea	0.00 (0.00, 0.00)	0.00 (0.00, 0.00)	0.00 (0.00, 0.00)	2.16	0.34
Abdominal pain	0.00 (0.00, 33.33)^∗^	0.00 (0.00, 33.33)	0.00 (0.00, 33.33)	6.47	0.04
Buttock pain	0.00 (0.00, 33.33)	0.00 (0.00, 33.33)	0.00 (0.00, 33.33)	1.88	0.39
Bloating	0.00 (0.00, 33.33)	0.00 (0.00, 33.33)	0.00 (0.00, 33.33)	1.33	0.51
Dry mouth	33.33 (0, 33.33)^∗^	33.33 (0.00, 66.67)	33.33 (16.67, 66.67)	22.24	< 0.01
Hair loss	0.00 (0.00, 33.33)^∗^	33.33 (0.00, 66.67)	33.33 (0.00, 66.67)	26.16	< 0.01
Taste	33.33 (0.00, 66.67)	33.33 (0.00, 66.67)	33.33 (0.00, 66.67)	4.42	0.11
Flatulence	33.33 (0.00, 66.67)	33.33 (0.00, 66.67)	33.33 (0.00, 66.67)	2.8	0.25
Fecal incontinence	0.00 (0.00, 16.67)	0.00 (0.00, 0.00)	0.00 (0.00, 0.00)	0.09	0.96
Sore skin	0.00 (0.00, 0.00)	0.00 (0.00, 33.33)	0.00 (0.00, 33.33)	3.17	0.21
Embarrassment	0.00 (0.00, 0.00)	0.00 (0.00, 0.00)	0.00 (0.00, 0.00)	2.41	0.30
Stoma care problems	0.00 (0.00, 0.00)	0.00 (0.00, 0.00)	0.00 (0.00, 0.00)	2.87	0.24
Impotence (men)	0.00 (0.00, 0.00)	0.00 (0.00, 0.00)	0.00 (0.00, 0.00)	4.12	0.12
Dyspareunia (women)	0.00 (0.00, 0.00)	0.00 (0.00, 0.00)	0.00 (0.00, 0.00)	3.66	0.06
Composite symptom score	17.28 (12.58, 24.92)	17.9 (12.35, 25.08)	17.44 (11.81, 24.31)	1.21	0.55

*Note:* T1 represents the early stage of chemotherapy, T2 represents the middle stage of chemotherapy, T3 represents the late stage of chemotherapy, and “^∗^” indicates a statistically significant difference compared with the T3 period.

**Table 3:** Results of changes in body composition variables over time during chemotherapy in patients with CRC ($\overset{¯}{\chi }$ ± *S*).

Variable	T1	T2	T3	*F*/*χ*^2^	*p*
*BIA-body composition*					
Hydration	50.55 ± 8.00	50.87 ± 7.34	50.36 ± 8.16	1.13	0.33
Protein	8.92 ± 1.90	8.87 ± 2.07	8.94 ± 2.25	0.61	0.54
Inorganic salt	2.62 ± 0.51	2.63 ± 0.52	2.63 ± 0.52	0.44	0.64
Body fat	17.20 ± 5.88	17.55 ± 6.01	17.95 ± 6.14	4.78	0.01
Body fat percentage (BIA)	28.89 ± 8.66	29.27 ± 8.57	29.53 ± 8.68	4.09	0.13
Weight	58.97 ± 9.07	59.6 ± 9.26	60.36 ± 9.79	10.82	< 0.01
Skeletal muscle mass	22.31 ± 0.33	22.35 ± 0.36	22.31 ± 0.32	0.15	0.86
BMI	22.33 ± 2.95	22.57 ± 3.00	22.9 ± 3.29	10.01	< 0.01
Waist-to-hip fat ratio	0.80 ± 0.10	0.81 ± 0.10	0.82 ± 0.10	15.64	< 0.01
Basic metabolism	1392 (1251, 1499)	1388 (1270, 1500)	1388 (1276, 1516)	1.89^∗^	0.39
Visceral fat	7.55 ± 3.39	7.69 ± 3.42	7.94 ± 3.61	3.75	0.03

*Body circumference*					
Chest circumference	89.65 ± 6.91	89.95 ± 6.69	90.82 ± 7.00	14.62	< 0.01
Waist circumference	83.74 ± 8.24	84.37 ± 8.28	84.96 ± 8.62	6.85	< 0.01
Hip circumference	90.33 ± 6.18	90.92 ± 5.72	91.68 ± 6.06	10.50	< 0.01
Upper arm circumference	27.57 ± 2.81	27.83 ± 2.87	28.28 ± 3.01	14.07	< 0.01
Thigh circumference	48.28 ± 4.41	48.89 ± 4.25	49.68 ± 4.92	14.38	< 0.01
Waist-to-hip ratio	0.93 ± 0.07	0.93 ± 0.06	0.93 ± 0.06	0.15	0.86

*Muscle powe*r					
Grip strength	26.84 ± 7.83	26.38 ± 8.22	26.60 ± 8.05	1.15	0.32
30 s chair–stand test	12.74 ± 4.08	13.50 ± 4.56	13.62 ± 4.46	10.70	0.07

*Skinfold thickness*					
Triceps brachii skin fold thickness	13.57 ± 4.63	14.18 ± 4.87	14.78 ± 4.73	16.42	< 0.01
Shoulder skin fold thickness	20.06 ± 7.70	21.29 ± 7.59	21.82 ± 7.74	19.35	< 0.01
Abdominal skin fold thickness	21.36 ± 7.50	22.64 ± 7.46	22.85 ± 7.64	9.61	< 0.01
Anterior iliac skin fold thickness	15.02 ± 6.42	16.64 ± 6.74	17.10 ± 6.34	19.0	< 0.01
Body fat percentage (formula)	21.63 ± 6.71	22.58 ± 6.85	23.12 ± 6.70	21.10	< 0.01

*Note:* T1 is early chemotherapy, T2 is middle-stage chemotherapy, and T3 is late chemotherapy.

^∗^Does not meet the normality test, according to the generalized estimation model.

**Table 4:** Differential analysis of skeletal muscle mass during chemotherapy across subgroups of patient-reported trajectories of outcome development (kg, M (P25, P75)).

PROs track grouping		Skeletal muscle mass (kg)					
		T1	*H* (*P*)	T2	*H* (*P*)	T3	*H* (*P*)
Patient-reported overall health outcome trajectory subgroups	Moderate-decline group (G1)(*n* = 104)	20.00 (17.23, 24.50)^a^	17.17 (< 0.01)	19.65 (17.65, 24.00)^a^	20.54 (< 0.01)	19.85 (17.23, 24.15)^a^	11.11 (< 0.01)
	High-stable group (G2) (*n* = 57)	23.70 (21.00, 26.80)^b^		24.90 (20.85, 27.65)^b^		24.30 (20.75, 27.00)^b^	
	Moderate-growth group (G3) (*n* = 33)	23.20 (18.85, 27.80)^bc^		24.20 (18.75, 27.65)^bc^		24.00 (18.85, 27.90)^bc^	
	Low-decline group (G4) (*n* = 7)	25.30 (18.60, 29.10)^ab^		24.70 (18.10, 27.40)^ab^		23.80 (18.00, 25.60)^ab^	

Subgroups of patient-reported functional outcome trajectories	Low function-decline group (C1)(*n* = 21)	20.20 (18.15, 27.55)	2.87 (0.24)	19.60 (17.95, 26.20)	2.81 (0.25)	19.20 (17.80, 25.10)	5.2 (0.07)
	High function-growth group (C2)(*n* = 85)	23.20 (19.40, 25.60)		23.00 (19.40, 26.65)		23.50 (20.05, 26.40)	
	Moderate function-stable group (C3)(*n* = 95)	21.00 (17.40, 25.90)		21.00 (17.90, 26.20)		20.70 (17.90, 25.60)	

Subgroups of patient-reported symptom trajectories	Low symptom-decline group (c1)(*n* = 108)	23.15 (19.25, 26.60)	4.86 (0.09)	23.45 (19.40, 26.60)^a^	7.56 (0.02)	23.75 (19.83, 26.50)^a^	10.33 (< 0.01)
	High symptom-growth group (c2)(*n* = 18)	19.55 (16.95, 25.93)		18.10 (15.50, 25.38)^bc^		18.55 (16.28, 24.25)^bc^	
	Moderate symptom-stable group (c3)(*n* = 75)	20.60 (18.00, 25.40)		20.20 (18.30, 25.60)^b^		20.30 (18.20, 25.00)^b^	

*Note:* T1 is the early stage of chemotherapy, T2 is the middle stage of chemotherapy, and T3 is the late stage of chemotherapy; superscripts a, b, and c are the results of multiple comparisons between groups; the presence of one or more identical letters indicates that there is no statistically significant difference between the groups (*p* > 0.05), and the presence of a completely different letter indicates that there is a statistically significant difference between the groups (*p* < 0.05).

**Table 5:** Differences in body fat percentage during chemotherapy among subgroups of reported outcome development trajectories in different patients (%, M (P25, P75)).

PROs grouping		Body fat percentage (%)					
		T1	*H* (*P*)	T2	*H* (*P*)	T3	*H* (*P*)
Patient-reported overall health outcome trajectory subgroups	Moderate-decline group (G1)(*n* = 104)	21.1 (17.25, 26.70)	3.68 (0.30)	22.20 (17.95, 28.26)	2.10 (0.55)	22.58 (18.02, 28.45)	0.74 (0.86)
	High-stable group (G2) (*n* = 57)	18.22 (14.58, 25.77)		20.65 (15.55, 26.39)		22.10 (16.78, 28.05)	
	Moderate-growth group (G3) (*n* = 33)	21.71 (17.79, 24.58)		21.95 (18.89, 25.78)		22.50 (19.99, 27.81)	
	Low-decline group (G4) (*n* = 7)	23.31 (17.95, 30.93)		20.84 (17.51, 29.96)		19.55 (17.93, 29.96)	

Subgroups of patient-reported functional outcome trajectories	Low function-decline group (C1)(*n* = 21)	20.30 (17.01, 25.73)	2.13 (0.35)	20.84 (16.28, 26.67)	2.11 (0.35)	19.75 (16.82, 25.13)	3.27 (0.20)
	High function-growth group (C2)(*n* = 85)	19.22 (15.51, 25.95)		20.94 (16.43, 26.69)		22.24 (17.32, 27.39)	
	Moderate function-stable group (C3)(*n* = 95)	21.12 (17.55, 26.25)		22.23 (18.79, 28.41)		23.02 (18.93, 28.81)	

Subgroups of patient-reported symptom trajectories	Low symptom-decline group (c1)(*n* = 108)	20.49 (15.58, 26.08)	1.36 (0.51)	21.35 (16.31, 27.52)	3.75 (0.15)	22.44 (17.40, 28.21)	5.45 (0.07)
	High symptom-growth group (c2)(*n* = 18)	18.94 (16.39, 24.49)		18.81 (16.33, 23.79)		18.99 (14.93, 23.64)	
	Moderate symptom-stable group (c3)(*n* = 75)	21.08 (17.40, 26.79)		22.06 (19.31, 28.41)		23.67 (19.31, 28.81)	

## Data Availability

The data that support the findings of this study are available from the corresponding author upon reasonable request.

## Supporting Information

Supporting Figure 1: Longitudinal follow-up flowchart of this study: A concise figure that includes descriptions of the loss of patients and reasons for loss at various time points during the longitudinal follow-up process.

Supporting Table 1: Calculation Methods for Neighborhood Scores of EORTC QLQ-C30 and EORTC QLQ-CR29: The dimensions and scoring methods of the patient-reported outcome scales (EORTC QLQ-C30 and EORTC QLQ-CR29) used in this study.

Supporting Tables 2-1–2-3 describe the relevant results of subgroup trajectory analysis with self-reported overall outcomes as the dependent variable, including fitting indicators for subgroup trajectories of overall outcomes, model parameters, and comparison of scores for four subgroup trajectories.

Supporting Table 2-1: Subgroup trajectory analysis of self-reported overall outcomes in patients with CRC.

Supporting Table 2-2: Estimated results of four subgroup model parameters for the development trajectory of self-reported overall outcomes by patients.

Supporting Table 2-3: Comparison of self-reported overall outcomes in patients with CRC undergoing chemotherapy on three different time scales.

Supporting Tables 3-1–3-3 describe the relevant results of subgroup trajectory analysis with self-reported functional outcomes as the dependent variable, including the fitting indicators, model parameters, and comparison of scores for three functional subgroup trajectories.

Supporting Table 3-1: Subgroup trajectory analysis of patient-reported functional outcome trajectories in patients with CRC.

Supporting Table 3-2: Estimated results of three subgroup model parameters for the functional development trajectory reported by patients.

Supporting Table 3-3: Differences in self-report function scores among three subgroups of patients with CRC undergoing chemotherapy.

Supporting Tables 4-1–4-3 describe the relevant results of subgroup trajectory analysis with self-reported symptom outcomes as the dependent variable, including the fitting indicators, model parameters, and comparison of scores for three symptom subgroup trajectories.

Supporting Table 4-1: LCGM/LGMM model fitting index for the total score of self-reported symptom neighborhoods in patients with CRC undergoing chemotherapy.

Supporting Table 4-2: Estimated results of three subgroup model parameters for symptom development trajectory reported by patients.

Supporting Table 4-3: Comparison of self-reported symptom scores among three subgroups of patients with CRC undergoing chemotherapy.
