# Supplementary material for: Patient-Reported Outcomes and Body Composition Changes in Patients With Colorectal Cancer During Chemotherapy: A Longitudinal Study
Source: J Nurs Manag. 2025 Aug 24;2025:1268096. doi: 10.1155/jonm/1268096 (PMC12399355; doi:10.1155/jonm/1268096)
Supplement: Supporting Information — Additional supporting information can be found online in the Supporting Information section. [file 1268096.f1.docx]

**Supplementary material documents**

**Supplementary Figure 1** Longitudinal follow-up flowchart of this study: A concise figure that includes descriptions of the loss of patients and reasons for loss at various time points during the longitudinal follow-up process.

**Supplementary Table 1** Calculation Methods for Neighborhood Scores of EORTC QLQ-C30 and EORTC QLQ-CR29: The dimensions and scoring methods of the patient reported outcome scales (EORTC QLQ-C30 and EORTC QLQ-CR29) used in this study.

**Supplementary Tables 2-1 to 2-3** describe the relevant results of subgroup trajectory analysis with self-reported overall outcomes as the dependent variable, including fitting indicators for subgroup trajectories of overall outcomes, model parameters, and comparison of scores for four subgroup trajectories.

**Supplementary Table 2-1** Subgroup trajectory analysis of self-reported overall outcomes in patients with CRC

**Supplementary Table 2-2** Estimated results of four subgroup model parameters for the development trajectory of self-reported overall outcomes by patients

**Supplementary Table 2-3** Comparison of self-reported overall outcomes in patients with colorectal cancer undergoing chemotherapy on three different time scales

**Supplementary Tables 3-1 to 3-3** describe the relevant results of subgroup trajectory analysis with self-reported functional outcomes as the dependent variable, including the fitting indicators, model parameters, and comparison of scores for three functional subgroup trajectories

**Supplementary Table 3-1** Subgroup trajectory analysis of patient-reported functional outcome trajectories in patients with CRC

**Supplementary Table 3-2** Estimated results of three subgroup model parameters for the functional development trajectory reported by patients

**Supplementary Table 3-3** Differences in self-report function scores among three subgroups of patients with colorectal cancer undergoing chemotherapy

**Supplementary Tables 4-1 to 4-3** describe the relevant results of subgroup trajectory analysis with self-reported symptom outcomes as the dependent variable, including the fitting indicators, model parameters, and comparison of scores for three symptom subgroup trajectories

**Supplementary Table 4-1** LCGM/LGMM model fitting index for the total score of self-reported symptom neighborhoods in patients with CRC undergoing chemotherapy

**Supplementary Table 4-2** Estimated results of three subgroup model parameters for symptom development trajectory reported by patients

**Supplementary Table 4-3** Comparison of self-reported symptom scores among three subgroups of patients with colorectal cancer undergoing chemotherapy


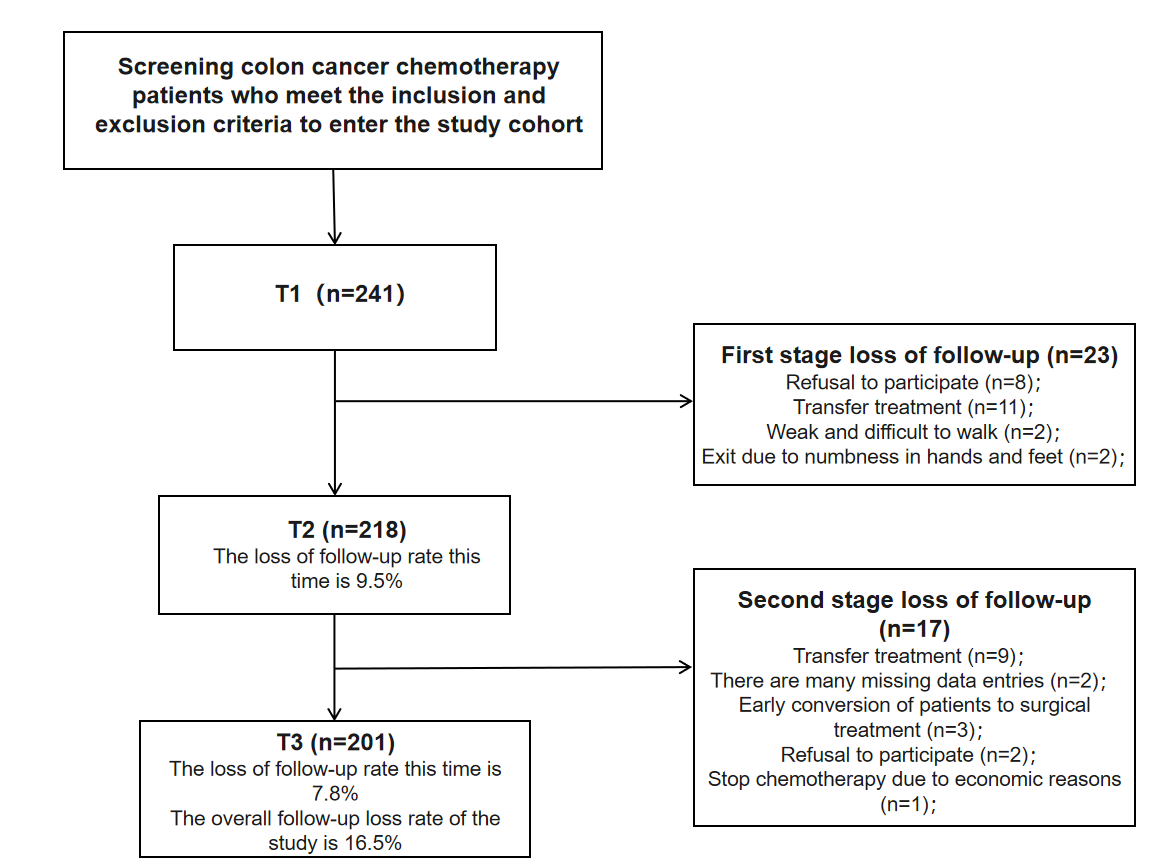


**Supplementary Figure 1** Longitudinal follow-up flowchart of this study: A concise figure that includes descriptions of the loss of patients and reasons for loss at various time points during the longitudinal follow-up process.

**Supplementary Table 1 Calculation Methods for Neighborhood Scores of EORTC QLQ-C30 and EORTC QLQ-CR29**

| dimension | abbreviated code | Attribute | number of items | Range (R) | Rough score (RS) |
| --- | --- | --- | --- | --- | --- |
| **EORTC QLQ-C30** |  |  |  |  |  |
| Physical Functioning | PF | functional type | 5 | 3 | （Q1+Q2+Q3+Q4+Q5）/5 |
| Role Functioning | RF | functional type | 2 | 3 | (Q6+Q7)/2 |
| Emotional Functioning | EF | functional type | 4 | 3 | (Q21+Q22+Q23+Q24)/4 |
| Cognitive Functioning | CF | functional type | 2 | 3 | (Q20+Q25)/2 |
| Social Functioning | SF | functional type | 2 | 3 | (Q26+Q27)/2 |
| Fatigue | FA | Symptom type | 3 | 3 | (Q10+Q12+Q18)/3 |
| Nausea / Vomiting | NV | Symptom type | 2 | 3 | (Q14+Q15)/2 |
| Pain | PA | Symptom type | 2 | 3 | (Q9+Q19)/2 |
| Dyspnoea | DY | Symptom type | 1 | 3 | Q8 |
| Sleep disturbance | SL | Symptom type | 1 | 3 | Q11 |
| Appetite loss | AP | Symptom type | 1 | 3 | Q13 |
| Constipation | CO | Symptom type | 1 | 3 | Q16 |
| Diarrhoea | DI | Symptom type | 1 | 3 | Q17 |
| Financial Problems | FI | Symptom type | 1 | 3 | Q28 |
| **Global** **health status** | QL | Quality of life | 2 | 6 | (Q29+Q30)/2 |
| **EORTC QLQ-CR29** |  |  |  |  |  |
| Body image | BI | functional type | 3 | 3 | （Q45+Q46+Q47）/3 |
| Anxiety | ANX | functional type | 1 | 3 | Q43 |
| Weight | WEI | functional type | 1 | 3 | Q44 |
| Sexual interest | SEXF | functional type | 2 | 3 | (Q56+Q58)/2 |
| Urinary frequency | UF | Symptom type | 2 | 3 | (Q31+Q32)/2 |
| Blood and mucus in Stool | BMS | Symptom type | 2 | 3 | (Q38+Q39)/2 |
| Stool frequency | SF2 | Symptom type | 2 | 3 | (Q52+Q53)/2 |
| Urinary incontinence | UI | Symptom type | 1 | 3 | Q33 |
| Dysuria | DY2 | Symptom type | 1 | 3 | Q34 |
| Abdominal Pain | AP2 | Symptom type | 1 | 3 | Q35 |
| Buttock Pain | BP | Symptom type | 1 | 3 | Q36 |
| Bloating | BF | Symptom type | 1 | 3 | Q37 |
| Dry mouth | DM | Symptom type | 1 | 3 | Q40 |
| Hair loss | HL | Symptom type | 1 | 3 | Q41 |
| Taste | TA | Symptom type | 1 | 3 | Q42 |
| Flatulence | FL | Symptom type | 1 | 3 | Q49 |
| Faecal incontinence | FI2 | Symptom type | 1 | 3 | Q50 |
| Sore skin | SS | Symptom type | 1 | 3 | Q51 |
| Embarrassment | EMB | Symptom type | 1 | 3 | Q54 |
| Stoma care problems | STO | Symptom type | 1 | 3 | Q55 |
| Impotence (male) | IMP | Symptom type | 1 | 3 | Q57 |
| Dyspareunia (women) | DYS | Symptom type | 1 | 3 | Q59 |

Functional field: SS = [1-(RS-1/R]×100;Symptom domain and general health domain standardized calculation: SS = [(RS-1)/R]×100.

**Supplementary Table 2-1 Subgroup trajectory analysis of** **self-reported overall outcomes in patients with CRC**

| Class | AIC | BIC | Entropy | LMR-LRT | BLRT | Class headcount ratio |
| --- | --- | --- | --- | --- | --- | --- |
| LCGM |  |  |  |  |  |  |
| CLASS-1 | 5259.12 | 5275.64 | - | - | - | - |
| CLASS-2 | 5133.12 | 5159.54 | 0.76 | ＜0.01 | ＜0.01 | 57.4%/42.5% |
| CLASS-3 | 5117.77 | 5154.11 | 0.83 | 0.52 | ＜0.01 | 4.2%/40.3%/ 55.5% |
| **CLASS-4** | 5102.35 | 5148.60 | 0.77 | ＜0.01 | ＜0.01 | 51.7%/28.4%/16.4%/3.5% |
| CLASS-5 | 5100.51 | 5156.67 | 0.72 | 0.68 | 0.10 | 18.9%/3.3%/19.4%/24.3%/34.1% |
| CLASS-6 | 5100.45 | 5166.52 | 0.74 | 0.12 | 0.35 | 20.3%/8.7%/32.9%/17.7%/16.4%/3.9% |
| LGMM |  |  |  |  |  |  |
| CLASS-1 | 5104.41 | 5130.83 | - | - | - | - |
| CLASS-2 | 5100.98 | 5137.32 | 0.60 | 0.05 | 0.10 | 54.4%/45.6% |
| CLASS-3 | 5104.62 | 5150.87 | 0.59 | 0.73 | 0.75 | 19.5%/54.07%/26.41% |
| CLASS-4 | 5102.69 | 5158.85 | 0.74 | 0.25 | 0.40 | 2.6%/0.1%/15.7%/84.6% |
| CLASS-5 | 5100.17 | 5166.23 | 0.72 | 0.32 | 0.10 | 22.5%/3.2%/30.7%/25.6%/17.9% |
| CLASS-6 | 5102.98 | 5178.96 | 0.72 | 0.54 | 0.65 | 32.2%/11.0%/17.2%/3.8%/20.0%/15.7% |

**Supplementary Table 2-2 Estimated results of four subgroup model parameters for the development trajectory of self-reported overall outcomes by patients**

| Patient-reported overall health outcome trajectory subgroups |  |  | Estimate  （‾*χ* ） | standard error（*SE*） | *t* | *P* |
| --- | --- | --- | --- | --- | --- | --- |
| Moderate-decline group (G1) | Mean | intercept | 44.45 | 1.73 | 25.64 | ＜0.01 |
|  |  | slope | -4.52 | 1.03 | -4.39 | ＜0.01 |
| High-stable group (G2) | Mean | intercept | 70.37 | 2.30 | 30.62 | ＜0.01 |
|  |  | slope | -2.27 | 1.35 | -1.69 | 0.09 |
| Moderate-growth group (G3) | Mean | intercept | 43.01 | 4.46 | 9.63 | ＜0.01 |
|  |  | slope | 8.97 | 3.13 | -2.86 | ＜0.01 |
| Low-decline group(G4) | Mean | intercept | 22.38 | 5.21 | 4.29 | ＜0.01 |
|  |  | slope | -4.53 | 2.35 | -1.92 | 0.04 |

**Supplementary Table 2-3 Comparison of self-reported overall outcomes in patients with colorectal cancer chemotherapy on three different time** **scales**

| item | Moderate-decline group (G1) （n=104） | High-stable group (G2) （n=57） | Moderate-growth group (G3) （n=33） | Low-decline group(G4) （n=7） | H | P |
| --- | --- | --- | --- | --- | --- | --- |
| health status score(T1) | 41.67(33.33,58.33)^a^ | 66.67(58.33,83.33)^b^ | 41.67(33.33,50.00)^ac^ | 25.00(16.67,25.00)^c^ | 76.72 | ＜0.01 |
| health status score(T2) | 33.33(33.33,41.67)^a^ | 66.67(58.33,75.00)^b^ | 50.00(41.67,58.33)^c^ | 16,67(8.33,25.00)^cd^ | 103.40 | ＜0.01 |
| health status score(T3) | 33.33(33.33,41.67)^a^ | 66.67(58.33,75.00)^b^ | 66.67(58.33,70.83)^bc^ | 8.30(8.30,16.67)^ac^ | 143.74 | ＜0.01 |

Note: T1 is the early stage of chemotherapy, T2 is the middle stage of chemotherapy, and T3 is the late stage of chemotherapy. a, b, c, and d are the results of multiple comparisons between groups. One or more identical letters indicate no statistical difference between groups (P > 0.05), while completely different letter marks indicate statistical difference between groups (P<0.05).

**Supplementary table 3-1 Subgroup trajectory analysis of Patient-reported functional outcome trajectories in patients with CRC**

| Class | AIC | BIC | Entropy | LMR-LRT | BLRT | Class headcount ratio |
| --- | --- | --- | --- | --- | --- | --- |
| LCGM |  |  |  |  |  |  |
| CLASS-1 | 4828.25 | 4844.77 | - | - | - | - |
| CLASS-2 | 4719.95 | 4746.38 | 0.69 | ＜0.01 | ＜0.01 | 47%/52% |
| **CLASS-3** | 4672.83 | 4709.17 | 0.83 | ＜0.01 | ＜0.01 | 10.4%/42.3%/ 47.3% |
| CLASS-4 | 4664.95 | 4711.20 | 0.86 | 0.16 | ＜0.01 | 7%/43.2%/46.7%/3.0% |
| CLASS-5 | 4661.98 | 4718.14 | 0.78 | 0.48 | 0.10 | 3.0%/18.9%/42.3%/7.4%/28.4% |
| CLASS-6 | 4664.73 | 4740.70 | 0.80 | 0.21 | 0.50 | 32.4%/9.5%/8.5%/2.5%/3.5%/43.8% |
| LGMM |  |  |  |  |  |  |
| CLASS-1 | 4719.95 | 4746.38 | - | - | - | - |
| CLASS-2 | 4674.46 | 4710.79 | 0.75 | 0.09 | ＜0.01 | 18.4%/81.5% |
| CLASS-3 | 4667.44 | 4713.69 | 0.82 | 0.10 | 0.10 | 11%/44%/44% |
| CLASS-4 | 4664.43 | 4720.59 | 0.84 | 0.23 | 0.10 | 44.8%/1.0%/44.3%/10.0% |
| CLASS-5 | 4662.72 | 4728.78 | 0.77 | 0.62 | 0.25 | 44%/32.3%/2.0%/8.5%/12.9% |
| CLASS-6 | 4661.43 | 4727.50 | 0.80 | 0.22 | 0.20 | 9.0%/2.5%/31.8%/41.8%/9.9%/5.0% |

**Supplementary table 3-2 Estimated results of three subgroup model parameters for the functional development trajectory reported by patients**

| Subgroups of Patient-reported functional outcome trajectories |  |  | Estimate  （‾*χ* ） | standard error（*SE*） | *t* | *P* |  |
| --- | --- | --- | --- | --- | --- | --- | --- |
| Low function-decline group (C1) | Mean | intercept | 60.51 | 3.59 | 16.86 | ＜0.01 |  |
|  |  | slope | -7.60 | 2.18 | -3.49 | ＜0.01 |  |
| High function-growth group (C2) | Mean | intercept | 77.62 | 1.40 | 55.56 | ＜0.01 |  |
|  |  | slope | 2.23 | 0.71 | 3.14 | ＜0.01 |  |
| Moderate function-stable group (C3) | Mean | intercept | 66.30 | 1.23 | 54.13 | ＜0.01 |  |
|  |  | slope | -0.17 | 0.90 | -0.19 | 0.85 |  |

**Supplementary table 3-3 Differences in self-report function scores among three subgroups of colorectal cancer chemotherapy patients**

| item | Low function-decline group (C1) （n=21） | High function-growth group (C2) (n=85) | Moderate function-stable group (C3) (n=95) | H | P |
| --- | --- | --- | --- | --- | --- |
| Functional score(T1) | 60.33(47.00,71.28)^a^ | 78.11(71.94,85.56)^b^ | 67.28(59.92,74.97)^c^ | 43.33 | ＜0.01 |
| Functional score(T2) | 50.44(46.39,60.83)^a^ | 80.67(73.56,85.17)^b^ | 64.83(57.36,68.39)^c^ | 108.26 | ＜0.01 |
| Functional score(T3) | 42.44(39.33,50.17)^a^ | 81.83(79.11,85.50)^b^ | 66.83(61.61,70.89)^c^ | 154.79 | ＜0.01 |

**Supplementary table 4-1 LCGM/LGMM model fitting index for the total score of self-reported symptom neighborhoodsin patients with CRC during chemotherapy**

| Class | AIC | BIC | Entropy | LMR-LRT | BLRT | Class headcount ratio |
| --- | --- | --- | --- | --- | --- | --- |
| LCGM |  |  |  |  |  |  |
| CLASS-1 | 4389.10 | 4405.62 | - | - | - | - |
| CLASS-2 | 4213.15 | 4239.58 | 0.82 | ＜0.01 | ＜0.01 | 73.6%/26.3% |
| **CLASS-3** | **4157.95** | **4194.29** | **0.81** | ＜0.01 | ＜0.01 | **53.7%/9.0%/ 37.3%** |
| CLASS-4 | 4140.20 | 4186.45 | 0.85 | 0.10 | ＜0.01 | 54.7%/33.3%/9.0%/3.0% |
| CLASS-5 | 4113.24 | 4169.40 | 0.82 | 0.02 | ＜0.01 | 3.0%/28.4%/18.9%/42.8%/7.0% |
| CLASS-6 | 4111.21 | 4177.28 | 0.84 | 0.45 | 0.15 | 42.3%/3.0%/18.9%/27.9%/4%/6% |
| LGMM |  |  |  |  |  |  |
| CLASS-1 | 4148.62 | 4175.05 | - | - | - | - |
| CLASS-2 | 4123.91 | 4160.25 | 0.90 | 0.07 | ＜0.01 | 91.5%/8.5% |
| CLASS-3 | 4115.94 | 4162.19 | 0.82 | 0.17 | ＜0.01 | 78.6%/3.5%/17.9% |
| CLASS-4 | 4107.94 | 4164.10 | 0.83 | 0.16 | 0.17 | 80.6%/10.0%/6.5%/3.0% |
| CLASS-5 | 4105.63 | 4171.69 | 0.78 | 0.72 | 0.40 | 27.8%/3.5%/10.9%/52.7%/5.0% |
| CLASS-6 | 4106.11 | 4182.09 | 0.80 | 0.11 | 0.35 | 3.5%/13.9%/51.2%/5%/1.5%/24.9% |

**Supplementary table 4-2 Estimated results of three subgroup model parameters for symptom development trajectory reported by patients**

| Subgroups of patient-reported symptom trajectories |  |  | Estimate  （‾*χ* ） | standard error（*SE*） | | *t* | *P* |
| --- | --- | --- | --- | --- | --- | --- | --- |
| Low symptom-decline group (c1) | Mean | intercept | 13.98 | 0.76 | 18.42 | | ＜0.01 |
|  |  | slope | -0.57 | 0.40 | -1.43 | | 0.15 |
| High symptom-growth group (c2) | Mean | intercept | 34.80 | 2.382 | 14.61 | | ＜0.01 |
|  |  | slope | 0.94 | 1.131 | 0.83 | | 0.41 |
| Moderate symptom-stable group (c3) | Mean | intercept | 23.27 | 1.19 | 19.50 | | ＜0.01 |
|  |  | slope | -0.02 | 0.65 | -0.03 | | 0.98 |

**Supplementary table 4-3 Comparison of self-reported symptom scores among three subgroups of colorectal cancer chemotherapy patients**

| item | Low symptom-decline group (c1)（n=108） | High symptom-growth group (c2) (n=18) | Moderate symptom-stable group (c3) (n=75) | H | P |
| --- | --- | --- | --- | --- | --- |
| Symptom score(T1) | 13.35(9.99,16.90)^a^ | 34.41(26.66,39.27)^b^ | 22.38(17.90,26.47)^c^ | 69.43 | ＜0.001 |
| Symptom score(T2) | 13.04(9.26,16.86)^a^ | 36.57(34.57,40.78)^b^ | 23.15(20.22,27.47)^c^ | 106.14 | ＜0.001 |
| Symptom score(T3) | 12.73(9.14,16.40)^a^ | 36.34(33.60,39.24)^b^ | 25.0(21.14,30.71)^c^ | 103.45 | ＜0.001 |

Note: T1 is the early stage of chemotherapy, T2 is the middle stage of chemotherapy, and T3 is the late stage of chemotherapy. a, b, c were the results of multiple comparisons between groups, and different letter marks indicated statistical differences between groups (P<0.05).
